# Supplementary material for: The paradoxical decline and growth of trust as a function of borderline personality disorder trait count: Using discontinuous growth modelling to examine trust dynamics in response to violation and repair
Source: PLoS One. 2020 Jul 23;15(7):e0236170. doi: 10.1371/journal.pone.0236170 (PMC7377394; doi:10.1371/journal.pone.0236170)
Supplement: S1 Table — (DOCX) [file pone.0236170.s002.docx]

| **S2 Table. Discontinuous Mixed-Effects Growth Models Predicting Trust as a Function of BPD Caseness after Controlling for Gender and Cognitive Reflective Ability** | | | | | | |
| --- | --- | --- | --- | --- | --- | --- |
|  | **Absolute** | | **Relative**  **(to preceding phase)** | | **Relative**  **(to formation phase)** | |
|  | **Est** | ***SE*** | **Est** | ***SE*** | **Est** | ***SE*** |
| Intercept | 48.97^***^ | 2.05 | 48.98^***^ | 2.06 | 49.00^***^ | 2.06 |
| Formation slope (FS) | 0.17 | 0.60 | 0.17 | 0.60 | 0.17 | 0.60 |
| Dissolution transition (DT) | -20.71^***^ | 2.49 | -20.88^***^ | 2.90 | -20.88^***^ | 2.90 |
| Dissolution slope (DS) | -1.50 | 1.20 | -1.67 | 1.31 | -1.67 | 1.32 |
| Restoration transition (RT) | 11.51^***^ | 1.93 | 13.01^***^ | 2.71 | -12.87^**^ | 4.28 |
| Restoration slope (RS) | 1.12^**^ | 0.41 | 2.61^*^ | 1.30 | 0.95 | 0.73 |
| Gender (Female) | -4.48^*^ | 2.14 | -4.49^*^ | 2.15 | -4.53^*^ | 2.15 |
| Cognitive reflective ability | 2.41^*^ | 1.04 | 2.40^*^ | 1.04 | 2.40^*^ | 1.04 |
| BPD caseness | 4.09 | 5.88 | 4.08 | 5.92 | 4.08 | 5.91 |
| FS * BPD caseness | -3.57 | 2.27 | -3.57 | 2.28 | -3.57 | 2.27 |
| DT * BPD caseness | -0.39 | 9.52 | 3.19 | 11.07 | 3.19 | 11.06 |
| DS * BPD caseness | 10.74^*^ | 4.59 | 14.32^**^ | 5.02 | 14.31^**^ | 5.03 |
| RT * BPD caseness | -14.08^†^ | 7.34 | -24.82^*^ | 10.34 | 21.32 | 16.34 |
| RS * BPD caseness | 2.45 | 1.57 | -8.29^†^ | 4.96 | 6.02^*^ | 2.80 |
| †*p* < .10, **p* < .05, ***p* < .01, ****p* < .001, tests are two-tailed, *N*=234 participants (*n*=16 ‘caseness’, *n*=218 ‘noncaseness’), 3510 observations. BPD caseness = 7 or more BPD traits reported on MSI-BPD. Cognitive reflective ability was *z*-standardized and centred at the sample mean. | | | | | | |
