# Supplementary material for: The paradoxical decline and growth of trust as a function of borderline personality disorder trait count: Using discontinuous growth modelling to examine trust dynamics in response to violation and repair
Source: PLoS One. 2020 Jul 23;15(7):e0236170. doi: 10.1371/journal.pone.0236170 (PMC7377394; doi:10.1371/journal.pone.0236170)
Supplement: Attachment — Submitted filename: Response to Reviewers.docx [file pone.0236170.s003.docx]

**The paradoxical decline and growth of trust as a function of borderline personality disorder trait count: Using discontinuous growth modelling to examine trust dynamics in response to violation and repair**

**Journal Requirements:**

*1. Please ensure that your manuscript meets PLOS ONE's style requirements, including those for file naming.*

AUTHORS’ RESPONSE: We have reviewed the manuscript and edited it to meet PLOS ONE’s style requirements including double spacing the paragraphs. We also edited Table 1 (P. 17) so that instead of using a landscape orientation, we used a regular portrait orientation and allowed the table to extend beyond the manuscript margins as described in the PlOS ONE Table formatting guidelines *(“Tables do not have strict width and height requirements. Do not split your table or otherwise try to make the table appear within the manuscript margins if it does not fit on one page”*).

*2. We note that you have provided funding information that is not currently declared in your Funding Statement. However, funding information should not appear in the Acknowledgments section or other areas of your manuscript. We will only publish funding information present in the Funding Statement section of the online submission form.*

*Please remove any funding-related text from the manuscript and let us know how you would like to update your Funding Statement.*

AUTHORS’ RESPONSE: As instructed we have removed the funding-related text from the manuscript (P. 37).

Please update the Funding Statement with the following:

*“This research received financial funding by the University of Wollongong. The funders had no role in study design, data collection and analysis, decision to publish, or preparation of the manuscript.”*

*3. Please amend your list of authors on the manuscript to ensure that each author is linked to an affiliation. Authors’ affiliations should reflect the institution where the work was done (if authors moved subsequently, you can also list the new affiliation stating “current affiliation:….” as necessary).*

AUTHORS’ RESPONSE: Since submitting the manuscript, one of our author’s has a new affiliation. We have made the relevant changes in the manuscript (P. 1).

**Reviewer #1 Comments**

*Overall:  I found this article interesting, compelling, and a provocative addition to the literature on trust in BPD. The authors used a 15 round trust game with variations in the amount that trustees returned during different phases of the game. Paradoxically, BPD severity was associated with declining trust when interacting with a new and cooperative partner, but increasing trust after multiple instances of trust violation by that partner. Higher BPD severity also predicted fast restoration of trust than originally formed. It is difficult to understand why a person would respond in this way, mistrusting benevolence and trusting malevolence, which increases the appeal of the study. The study is supported by a thoughtful research design which is further enhanced by the use a sophisticated statistical approach. The study limitations have been well supported. I would like to see this study replicated with a clinical sample.*

AUTHORS’ RESPONSE: We are very glad that Reviewer 1 found this study interesting, compelling, and provocative, the design thoughtful and the data analysis sophisticated. We find the suggestion of replicating this study with a clinical sample very important and view our findings as a compelling argument for future research using clinical samples to examine trust in BPD as a dynamic construct.

*1. I don’t really see the need for the BPD categorical analyses, particularly given the small number of cases. I don’t mind its inclusion, but particularly given these are not clinician diagnoses, the dimensional approach seems superior.*

AUTHORS’ RESPONSE: We agree that the dimensional approach is superior given our sample characteristics. Although we have not used a clinical sample, we chose to include the categorical analyses to provide additional support for the potential clinical utility of our findings. We view this as providing a bridge to future research with clinical samples. Having said that, if reviewer 1 wishes, we can easily remove this supplemental analysis from the manuscript.

*2. I wonder if the findings could be interpreted through an interpersonal/attachment lens. People with BPD tend to oscillate in terms of interpersonal and attachment style, withdrawing when connection is available and approach when it is not. Once the partner returned to cooperative play, people with higher BPD again withdraw. This mirrors the tendency of patient with BPD to show severe mistrust of clinicians and at the same time significant trust towards sometimes exploitative and dangerous romantic partners.*

AUTHORS’ RESPONSE: Thank you for these suggestions. We agree that these findings may be interpreted through an attachment framework as outlined in the discussion (P. 36). While previous research has looked at the effect of attachment style on trust behaviours [e.g., 1, 2, 3], it would indeed be very interesting to examine whether the patterns of oscillations in trust that we observed in our high BPD trait individuals was underpinned by a fearful attachment style and/or other interpersonal factors. In fact, we are currently in the process of examining the effect of other interpersonally relevant variables including attachment characteristics on trust behaviour in another study. Your observation that the trust patterns we observed mirror interpersonal behaviour in patients with BPD is a really important one, but there appears to be little research looking at the within-person variations in trust preferences. As mentioned in our discussion (P. 34), one exception is the finding that although patients with BPD do not differ from healthy controls in their perception of fairness of partners in an economic game, they are more likely to have a preference to interact with unfair over fair partners, while the opposite preference is observed in healthy controls [4]. This is consistent with our own finding that high BPD-trait individuals appear to be more trusting when their partner is being less cooperative. It is certainly important to investigate this phenomenon given that maintenance of stable and enduring interpersonal relationships is associated with recovery in BPD [5].

*3. To align with previous literature, I have most typically seen the player who invests money in the trustee called the 'investor' rather than the 'trustor'.*

AUTHORS’ RESPONSE: Thank you for bringing this to our attention. We have also found that the terms ‘trustor’ and ‘investor’ have been used interchangeably across disciplines. To align with the BPD trust game literature, we have edited the manuscript replacing ‘trustor’ with ‘investor,’ as suggested. Replacements were made on pages 4, 6, 10-13, 25-26, 32. We also changed the terminology in the Supporting Information S1 Dataset.

**Reviewer #2 Comments**

*1. It seems that a ratio of 33-44% is rather low, since with 33% the trustor would receive only the amount he/she invested before. One may ask whether this can really be regarded as a fair behaviour of the trustee.*

AUTHORS’ RESPONSE: Thank you for this comment. It has highlighted a need for further clarity around the reciprocity ranges we selected. We based the reciprocity range of 34-44% for formation and restoration on various sources. A meta-analysis of 162 studies found mean trustee repayments approximated 37% [6]. This is similar to the reciprocity ratios of healthy trustees in the BPD literature [7]. In order to ensure an average reciprocity within the formation and restoration phases that was comparable to previous research, we needed to select a range that would ensure the investor did not lose any money, but also did not receive unrealistically high returns. We also needed to provide a wide enough range that there would be sufficient variability for randomization reducing the risk of participants receiving the same reciprocity ratios for each round. By choosing a range of 34-44%, we were able to achieve an average trustee reciprocity ratio across our sample of 38.96 (SD = 1.51) for formation and 38.99 (SD =1.22) for restoration, indicating that overall, investors profited during those phases.

To provide greater clarity on why the reciprocity ratios we chose are appropriate for this study, we have added the following statement to page 12 of the manuscript:

*“A trustee reciprocity range of 34-44% was selected to ensure repayments would be comparable to the reciprocity ratios observed in human trustees in previous research [16, 46].”*

*2. Please provide more information on how this was done (is it possible that single participants received in all trials only 33% repay ?).*

AUTHORS’ RESPONSE: The reciprocity ratios were randomly allocated within the specified ranges for each round per participant by a computer algorithm. Specifically, the trustee was programmed to return 34%-44% in the formation phase, 0%-10% in the dissolution phase, and 34%-44% in the restoration phase. To illustrate this, Fig 1 plots the reciprocity ratios for the first 12 participants. Visual observation of the distributions emphasizes both the variation in reciprocity ratios within the programmed ranges, both within and between participants.

**Fig 1. Trustee Reciprocity Ratios per Round for 12 Participants.** Each circle represents the percentage of the trustor’s tripled investment, returned by the trustee.

We ran further analyses on our data to determine if any participant received the same reciprocity ratio within the three phases (formation, dissolution, and restoration). Table 1 lists the number of participants per phase by number of unique reciprocity ratios. For example, a unique reciprocity ratio of 1 would be if a participant was randomly allocated the same reciprocity ratio for every round of the phase (e.g. round 1 = 40%, round 2 = 40%, round 3 = 40%). A unique reciprocity ratio of 2 would reflect an allocation of at least 2 different reciprocity ratios across the phase (e.g., round 1 = 40%, round 2 = 40%, round 3 = 37%), and so on. The minimum number of unique reciprocity ratios a participant was allocated in the formation and restoration phases, was 2 and 3, respectively. Due to the dissolution phase only comprising three rounds, the chance for uniform reciprocity ratios was higher. Indeed, we found that 3 participants were allocated uniform reciprocity ratios during this phase (reciprocity ratios of 1%, 7%, or 8%, respectively). All 3 participants scored in the Low BPD group so we were not concerned that the uniform reciprocity ratios could have influenced the direction of our findings.

| **Table 1. Number of participants per Unique Reciprocity Ratio during the Formation, Dissolution, and Restoration Phases.** | | | | | | | |
| --- | --- | --- | --- | --- | --- | --- | --- |
|  | Number of Unique Reciprocity Ratios | | | | | | |
|  | 1 | 2 | 3 | 4 | 5 | 6 | 7 |
| Formation | *n* = 0 | *n* = 12 | *n* = 85 | *n* = 137 | n/a | n/a | n/a |
| Dissolution | *n* = 3 | *n* = 58 | *n* = 173 | n/a | n/a | n/a | n/a |
| Restoration | *n* = 0 | *n* = 0 | *n* = 6 | *n* = 42 | *n* = 67 | *n* = 99 | *n* = 20 |

*3. Can the authors make sure that the repay ratios were comparable for participants in the three BPD groups in general, but also during the course of the different trials during formation and restoration? Please provide the corresponding data.*

AUTHORS’ RESPONSE:

Thank you for this suggestion, this is indeed a critical point. To respond this question, we first ran simple descriptive statistics on the trustee reciprocity ratios per round (Table 2). To test whether there were systematic differences in trustee reciprocity ratios are similar across respondents, regardless of BPD level, we conducted both correlational and ANOVA analyses.

| **Table 2. Means, Standard Deviations, and Intercorrelations of Trustee Reciprocity Ratios and BPD Trait Count per Round.** | | | |
| --- | --- | --- | --- |
| Round | Mean | *SD* | Correlation |
| 1 | 39.09 | 3.24 | -0.02 |
| 2 | 38.87 | 3.10 | 0.10 |
| 3 | 38.94 | 3.16 | -0.07 |
| 4 | 38.93 | 3.15 | 0.03 |
| 5 | 4.88 | 3.04 | 0.01 |
| 6 | 5.24 | 3.10 | -0.06 |
| 7 | 5.18 | 3.19 | 0.01 |
| 8 | 38.61 | 3.09 | -0.04 |
| 9 | 39.02 | 3.19 | -0.12 |
| 10 | 39.40 | 3.24 | -0.07 |
| 11 | 38.80 | 3.15 | -0.05 |
| 12 | 38.94 | 3.28 | -0.09 |
| 13 | 39.16 | 3.07 | -0.02 |
| 14 | 38.99 | 3.11 | -0.02 |
| BPD = BPD trait count (MSI-BPD), range 0-10 | | | |

First, we correlated BPD with reciprocity ratio per round. As seen in Table 2, all correlational effect sizes between trustee reciprocity ratio and BPD trait count were small [8] and found to be not statistically significant.

Next, we created three BPD categories based on respondent trait endorsement on the McLean Screening Instrument for Borderline Personality Disorder [MSI-BPD; 9] to conduct an ANOVA analysis. Using the MSI-BPD recommended cut-off for caseness, participants were categorized as High if they endorsed 7-10 MSI-BPD traits. Based on findings from the Collaborative Longitudinal Personality Disorders Study [CLPS: 10], 3 or more BPD traits is considered to indicate the presence of a notable level of borderline pathology, prompting us to categorize participants as Moderate if they endorsed 3-6 MSI-BPD traits. The remaining participants scoring 0-2 on the MSI-BPD were categorized as Low. Means and standard deviations for the trustee reciprocity ratios for each BPD category are presented in Table 3, while Fig’s 2a-c plot trustee reciprocity ratios for each phase as a function of BPD category.

| **Table 3. Means, Standard Deviations, and ANOVA of Trustee Reciprocity Ratios per Phase as a Function of BPD Category.** | | | | | | | |
| --- | --- | --- | --- | --- | --- | --- | --- |
|  | Low BPD (N=177) | | Moderate BPD (N=41) | | High BPD (N=16) | |  |
|  | Mean | *SD* | Mean | *SD* | Mean | *SD* | Kruskal Wallis Rank Test |
| BPD traits | 0.50 | 0.72 | 4.05 | 1.18 | 8.00 | 1.10 | - |
| Formation | 38.96 | 1.50 | 38.87 | 1.46 | 39.17 | 1.73 | *χ^2^*(2*)* = 0.41, *p* = 0.81 |
| Dissolution | 5.14 | 1.83 | 5.09 | 1.64 | 4.71 | 1.46 | *χ^2^*(2) = 0.64, *p* = 0.73 |
| Restoration | 39.09 | 1.21 | 38.55 | 1.17 | 38.96 | 1.20 | *χ^2^*(2) = 4.19, *p* = 0.12 |

| (a) Formation | (b) Dissolution | (c) Restoration |
| --- | --- | --- |
|  |  |  |
| **Fig 2. Trustee Reciprocity Ratios as a Function of Borderline Personality Disorder Category.** Low BPD are participants who endorsed 0-2 traits on the McLean Screening Instrument for Borderline Personality Disorder, while Moderate BPD participants endorsed 3-6 traits, and High BPD endorsed 7-10 traits. | | |

As there were significant discrepancies in the sample sizes across the BPD groups (*N_Low_* = 177; *N_Moderate_* = 41; and *N_High_* = 16) and evidence of non-normality in some distributions, we elected to run a non-parametric ANOVA analysis. The Kruskal-Wallis rank sum test did not show evidence for significant differences in trustee reciprocity ratios between BPD categories in any of the phases as outlined in Table 3.

To help provide greater clarity to readers, we added the following paragraph to the results section (P. 19):

“*To examine whether there were systematic differences in trustee reciprocity rates we conducted both correlation and ANOVA analyses so that we could treat BPD trait count as a continuous and categorical variable. We created three BPD categories based on number of traits endorsed on the MSI-BPD: Low (0-2), Moderate (3-6), and High (7-10). Due to significant discrepancies in sample size (N = 177/41/16, respectively) and evidence of non-normality and heterogeneity of variance in some distributions, we elected to run a non-parametric ANOVA analysis. Both, the correlation approach and the Kruskal–Wallis H Test did not show any significant difference in trustee reciprocity between BPD categories.*”

REFERENCES

1.	McClure MJ, Bartz JA, Lydon JE. Uncovering and overcoming ambivalence: The role of chronic and contextually activated attachment in two-person social dilemmas. J Pers. 2013;81(1):103-17. doi: 10.1111/j.1467-6494.2012.00788.x. PubMed PMID: 85018365.

2.	Bartz J, Simeon D, Hamilton H, Kim S, Crystal S, Braun A, et al. Oxytocin can hinder trust and cooperation in borderline personality disorder. Soc Cogn Affect Neurosci. 2011;6(5):556-63. doi: 10.1093/scan/nsq085.

3.	Taheri M, Rotshtein P, Beierholm U. The effect of attachment and environmental manipulations on cooperative behavior in the prisoner's dilemma game. PLoS One. 2018;13(11):e0205730. doi: 10.1371/journal.pone.0205730.

4.	Jeung H, Vollmann M, Herpertz SC, Schwieren C. Consider others better than yourself: Social decision-making and partner preference in borderline personality disorder. J Behav Ther Exp Psychiatry. 2018. doi: 10.1016/j.jbtep.2018.11.004. PubMed PMID: S0005791618302039.

5.	Zanarini MC, Frankenburg FR, Reich DB, Wedig MM, Conkey LC, Fitzmaurice GM. The course of marriage/sustained cohabitation and parenthood among borderline patients followed prospectively for 16 years. Journal Of Personality Disorders. 2015;29(1):62-70. doi: 10.1521/pedi_2014_28_147. PubMed PMID: 24963829.

6.	Johnson ND, Mislin AA. Trust games: A meta-analysis. J Econ Psychol. 2011;32(5):865-89. doi: 10.1016/j.joep.2011.05.007. PubMed PMID: S0167487011000869.

7.	King-Casas B, Sharp C, Lomax-Bream L, Lohrenz T, Fonagy P, Montague RR. The rupture and repair of cooperation in borderline personality disorder. Science. 2008;321(5890):806-10. doi: 10.1126/science.1156902.

8.	Cohen J. Statistical power analysis for the behavioral sciences. 2nd ed. ed. Hillsdale, NJ: Erlbaum; 1988.

9.	Zanarini MC, Vujanovic AA, Parachini EA, Boulanger JL, Frankenburg FR, Hennen J. A screening measure for BPD: The McLean Screening Instrument for Borderline Personality Disorder (MSI-BPD). Journal of Personality Disorders. 2003;17:568-73. doi: 10.1521/pedi.17.6.568.25355.

10.	Gunderson JG, Stout RL, McGlashan TH, Shea MT, Morey LC, Grilo CM, et al. Ten-year course of borderline personality disorder: psychopathology and function from the Collaborative Longitudinal Personality Disorders study. Arch Gen Psychiatry. 2011;68(8):827-37. doi: 10.1001/archgenpsychiatry.2011.37. PubMed PMID: 21464343.
